# Differences in multiple immune parameters between Indian and U.S. infants

**DOI:** 10.1371/journal.pone.0207297

**Published:** 2018-11-16

**Authors:** Deepak K. Rathore, Tyson H. Holmes, Kari C. Nadeau, Pratima Mittal, Achla Batra, Yael Rosenberg-Hasson, Shailaja Sopory, Rohit Gupta, Harish K. Chellani, Kailash C. Aggarwal, Vineeta Bal, Uma Chandra Mouli Natchu, Shinjini Bhatnagar, Morvarid Tavassoli, Deirdre J. Lyell, Satyajit Rath, Nitya Wadhwa, Holden T. Maecker

**Affiliations:** 1 Pediatric Biology Center, Translational Health Science & Technology Institute, NCR Biotech Science Cluster, 3rd Milestone, Faridabad-Gurgaon Expressway, Faridabad, Haryana, India; 2 Institute for Immunity, Transplantation, and Infection, Stanford University School of Medicine, Stanford, United States of America; 3 Division of Infectious Diseases, Department of Medicine, Stanford University School of Medicine, Stanford, United States of America; 4 Sean N. Parker Center for Allergy and Asthma Research at Stanford University; 5 Department of Obstetrics and Gynecology, Vardhman Mahavir Medical College and Safdarjung Hospital, New Delhi, India; 6 Department of Pediatrics, Vardhman Mahavir Medical College and Safdarjung Hospital, New Delhi, India; 7 National Institute of Immunology, New Delhi, India; 8 Department of Obstetrics and Gynecology, Stanford University School of Medicine, Stanford, United States of America; Monash University, AUSTRALIA

## Abstract

To compare immune phenotypes across two geographic and ethnic communities, we examined umbilical cord blood by flow cytometry and Luminex in parallel cohorts of 53 newborns from New Delhi, India, and 46 newborns from Stanford, California. We found that frequencies of a B cell subset suggested to be B-1-like, and serum IgM concentration were both significantly higher in the Stanford cohort, independent of differences in maternal age. While serum IgA levels were also significantly higher in the Stanford cohort, IgG1, IgG2, and IgG4 were significantly higher in the New Delhi samples. We found that neutrophils, plasmacytoid dendritic cells, CD8+ T cells, and total T cells were higher in the U.S. cohort, while dendritic cells, patrolling monocytes (CD14dimCD16+), natural killer cells, CD4+ T cells, and naïve B cells were higher in the India cohort. Within the India cohort, we also identified cell types whose frequency was positively or negatively predictive of occurrence of infection(s) in the first six months of life. Monocytes, total T cells, and memory CD4+ T cells were most prominent in having an inverse relationship with infection. We suggest that these data provide impetus for follow-up studies linking phenotypic differences to environmental versus genetic factors, and to infection outcomes.

## Introduction

Comparative immune phenotyping between different geographical and ethnic communities is largely lacking and could form the basis for better understanding of the unique disease burdens seen in different communities around the world. In particular, umbilical cord blood immune phenotypes are interesting to compare, since (a) they represent a very early phase of immunological development; (b) they are not influenced by post-birth environmental exposures which would likely increase the variability within a population; and (c) they may relate best to disease outcomes in the first months of life, which is when infection risk is greatest. Furthermore, cord blood is a readily available source of large numbers of immune cells and is usually discarded, making it a highly feasible tissue to study.

One major difference in global health outcomes is the burden of infections in neonatal life. At least some of these may be attributable to developmental differences in the immune system, which in turn could be due to *in utero* environmental differences, including, for example, toxin exposures, nutrition, and maternal infectious burden.

Circulating ‘natural’ antibodies as well as conventional T-dependent antibody responses are major protective determinants of neonatal mammalian health and are functionally immature in neonates and infants [1]. The state of responsiveness of the B cell compartment at birth, therefore, is of significant interest in understanding and addressing issues of vaccine efficacy as well as infection-related morbidity. Umbilical cord blood contains a substantial number of B lymphocytes; in fact, the numbers are greater than in adult blood; they increase over the first two years and then slowly decline to adult levels [2].

‘Natural’ antibodies are thought to be made by the sub-lineage of B-1 cells, which contribute an “innate-like” adaptive immune response by very rapidly secreting antibodies in response to antigen [3]. They have a repertoire for a broad spectrum of targets including both self-antigens and microbial pathogens [4] and are capable of self- renewal [5]. B-1 B cells are identified in the mouse immune system by expression of CD5 [6]. However, CD5 expression on human B cells has not been a reliable marker for the B-1 lineage [7]. Recently, there have been suggestions identifying human B-1 B cells in peripheral blood as being CD43+CD27+ [7], although there has been some controversy about this as well, with indications that this subset can likely include pre-plasmablasts and/or memory B cells [8–10]. The published frequency of CD43+CD27+ B-1 cells in umbilical cord blood for a U.S. cohort was lower than in adult blood, but not inordinately so [7].

The classical naïve B cells (or B-2 cells) emerge from the bone marrow. Immature B cells exiting the bone marrow have been identified in human peripheral blood principally by expression of the immaturity marker CD10 [11]. These cells are thought to be equivalent to the immature ‘transitional’ B cells seen in the mouse spleen and are poorly responsive to antigenic stimulation. As they mature, they lose CD10 expression and gain higher levels of CD22 and CD23 [11]. Clearly, the frequency of immature B cells in the cord blood would be expected to be a relevant marker for the state of maturity of the neonatal B cell compartment. In studies from the developed world, CD10+ B cell frequencies range from about 5% [12] to about 14% [13].

In addition to B cell subsets, differences in innate immune cells such as NK, monocyte and dendritic cell subsets could also influence disease protection in the newborn. And although T cells should be overwhelmingly naïve in umbilical cord blood, differentiation and/or activation of subsets of CD4+ and CD8+ T cells could also be indicators of immunological health in the newborn.

Our primary hypothesis was that differences in B-1-like cells between U.S. and Indian cord bloods could be related to their circulating IgM levels, and in turn, their post-natal infectious burden. Secondarily, we hypothesized that other cell types may differ between these cohorts and influence their resistance to post-natal infections. We therefore gathered data on multiple immune cell types and Ig isotypes in parallel cohorts of newborns from New Delhi, India, and Stanford, CA, USA.

## Materials and methods

### Study design

This parallel cohort study was undertaken from 2011–2016 at Vardhman Mahavir Medical College & Safdarjung Hospital (VMCC & SJH) in New Delhi India, and Lucile Packard Children’s Hospital at Stanford, CA in the US. The study protocol was approved by the respective institutional ethics committees (IEC) of VMMC & SJH and Lucile Packard Children’s Hospital, as well as the Translational Health Sciences and Technology Institute (THSTI) IEC. Written informed consent was obtained from interested mothers after explanation of the study. All samples and data were de-identified for analysis by the study team. Subject identifiers were kept only by the clinical teams in a secure database.

### Subject eligibility and assessment

Pregnant women were consented for the study if they were likely to deliver a singleton term baby vaginally and were likely to come for the 6-month follow up visit. Exclusion criteria included multiple gestation, any fetal congenital anomaly diagnosed in utero, a documented major medical or surgical illness, any pregnancy induced illness like gestational diabetes, pre-eclampsia, eclampsia, etc., a history of intake of immunomodulatory medications, history of blood transfusion or hospitalization due to an infection anytime during pregnancy, or any infection in the last trimester which lasted for three or more days. Pregnant women who had opted for cord blood ‘banking’ were also excluded. Newborns were assessed for ‘at-birth’ eligibility, to exclude those with one minute APGAR of **<**7/10, birth weight **<**1.8 kg, presence of Rh incompatibility or requirement for neonatal intensive care. If the newborn was also found eligible, cord blood was collected as described below.

Gestational age of the newborn was estimated based on date of ‘last menstrual period’. If this was not available, a dating ultrasound examination at the first visit was used to confirm gestational age. Newborns with a gestational age of > 37 weeks and up to 41 completed weeks were included in the study.

For the Indian site, mothers were asked to come at 24 weeks for follow up and morbidity data collection. If the mother was unable to come, the field assistant made a home visit to collect the data after taking permission from the mother and family members and at a time convenient for them.

### Cord blood collection and processing

Cord blood was collected from the cut end of the umbilical cord attached to the placenta, after the placenta was delivered. A sterile 20 G butterfly needle attached to a 10 ml syringe was inserted into the umbilical cord vein, with the bevel facing up, and 35+/-5 mL blood was collected by negative suction.

The entire fraction of blood was added immediately to a sterile specimen cup treated with sodium heparin for cryopreservation of mononuclear cells and plasma supernatant. The separated plasma was aliquoted into five 0.5 ml trace element-free vials and stored at -80°C.

To isolate mononuclear cells, g cord blood was diluted 1:1 with PBS, then underlaid with 1mL Ficoll for every 4 mL of diluted cord blood, followed by density gradient centrifugation and then treatment in RBC Lysis Buffer (Stem Cell Technologies, #07850) for removal of remaining erythrocytes. Resulting mononuclear cells were cryopreserved in 45% fetal bovine serum + 10% DMSO in RPMI-1640.

### Immunoglobulin analysis by Luminex

A panel of immunoglobulins (IgG1, IgG2, IgG3,I gG4, IgM and IgA) were analysed in a Luminex 200 instrument at the laboratories of the 2 sites using the same kit from MilliporeSigma (Burlington, MA, USA) and following a standardised protocol. The manufacturer’s directions were followed, except for two changes: (a) initial incubation of serum with beads was performed overnight at 4°C, and (b) serum was diluted 1:8000 rather than 1:16,000 as recommended in the protocol. Limits of detection (LODs, in ng/ml) were as follows: IgG2, lower LOD = 39.92 (US); IgG3, upper LOD = 2.3x10^6^ (India) and 12.4x10^6^ (US); IgG4, upper LOD = 4.2x10^6^ (India); IgA, lower LOD = 2.04 (India) and 2.06 (US).

### Flow cytometry

All flow cytometry was done at the Indian laboratory at THSTI, with 46 U.S. samples shipped to India as frozen cells. This helped in establishing equivalency in phenotyping at the two different sites. Cells were thawed at 37°C, counted, and resuspended for flow cytometry. The following pre-conjugated fluorescence labeled anti-human monoclonal antibodies (clones) were used: CD45 (2D1), CD16 (3G8), CD14 (M5E2), CD11c (B-ly6), CD3 (OKT-3), CD56 (NACAM16.2), CD4 (RPA-T4), CD25 (M-A251), CD127 (HIL-7R-M21), CD8 (RPA-T8), CD45RA (HI100), CCR7 (150503), CD19 (HIB19), CD27 (M-T271), CD24 (ML5), CD123 (7G3), Lineage-1 (SK7, 3G8, SJ25C1, L27, MφP9, NCAM16.2), TCR γ/δ (B1), (from BD Biosciences, San Jose, CA, USA) and CD66b (G10F5), CD163 (GHI/61), CD20 (2H7), CD10 (HI10a), CD43 (10G7), CD38 (HIT2), HLA-DR (L243), Vα24 Jα18 TCR (6B11) (from BioLegend Inc. San Diego, CA, USA). A 5-cocktail panel was used (antibody clones and fluorochromes are shown in Table 1). Each staining tube contained 1 million cells. Samples were incubated on ice for 30 min in the dark with pre-titrated optimal concentrations of monoclonal antibodies and washed with phosphate-buffered saline (PBS). Cells were fixed with 2% paraformaldehyde, washed, and 0.5 million cells from each sample acquired on a flow cytometer (FACSCanto II, BD Biosciences San Jose, CA). Cytometer Setup & Tracking (CST) beads to follow the instrument performance as a quality control parameter. Analysis was done using FlowJo software (TreeStar, Eugene, OR, USA).

**Table 1:** Cell-surface markers and monoclonal antibody cocktails.

Cocktail	Antibody (Clone)	Fluorochrome
A	CD45 (2D1)	APC-H7
	CD16 (3G8)	PE-Cy7
	CD66b (G10F5)	PerCP-Cy5.5
	CD14 (M5E2)	FITC
	CD11c (B-ly6)	BV 421
	CD163 (GHI/61)	APC
B	CD45 (2D1)	APC-H7
	CD3 (OKT-3)	PerCP-Cy5.5
	CD56 (NACAM16.2)	PE-Cy7
	CD4 (RPA-T4)	BV 421
	CD25 (M-A251)	FITC
	TCR γ/δ (B1)	PE
	Vα24 Jα18 TCR (6B11)	APC
	CD127 (HIL-7R-M21)	BV 510
C	CD45 (2D1) CD3 (HI3Ta)	APC-H7 BV 510
	CD4 (RPA-T4)	BV 421
	CD8 (RPA-T8)	PerCP-Cy5.5
	CD45RA (HI100)	PE-Cy7
	CCR7 (150503)	PE
	TCR γ/δ (B1)	FITC
	CD56 (NACAM16.2)	FITC
	CD25 (M-A251)	FITC
D	CD45 (2D1)	APC-H7
	CD19 (HIB19)	APC
	CD20 (2H7)	PerCP-Cy5.5
	CD10 (HI10a)	PE-Cy7
	CD43 (10G7)	FITC
	CD27 (M-T271)	BV 421
	CD38 (HIT2) CD24 (ML5)	PE BV 510
E	CD45 (2D1)	APC-H7
	Lineage 1 (SK7, 3G8, SJ25C1, L27, MøP9, NCAM16.2)	FITC
	HLADR (L243)	PE-Cy7
	CD11c (B-ly6)	BV 421
	CD123 (7G3)	APC

Based on the immunophenotyping panel of **Table 1**, we identified B-1 cells by first gating on CD3-CD19+ lymphocytes, then determined the proportion of these that were CD43+CD27+. We identified immature B-2 cells by similarly gating on CD3-CD19+ lymphocytes and determined the proportion of these that were CD10+.

**Fig 1:**
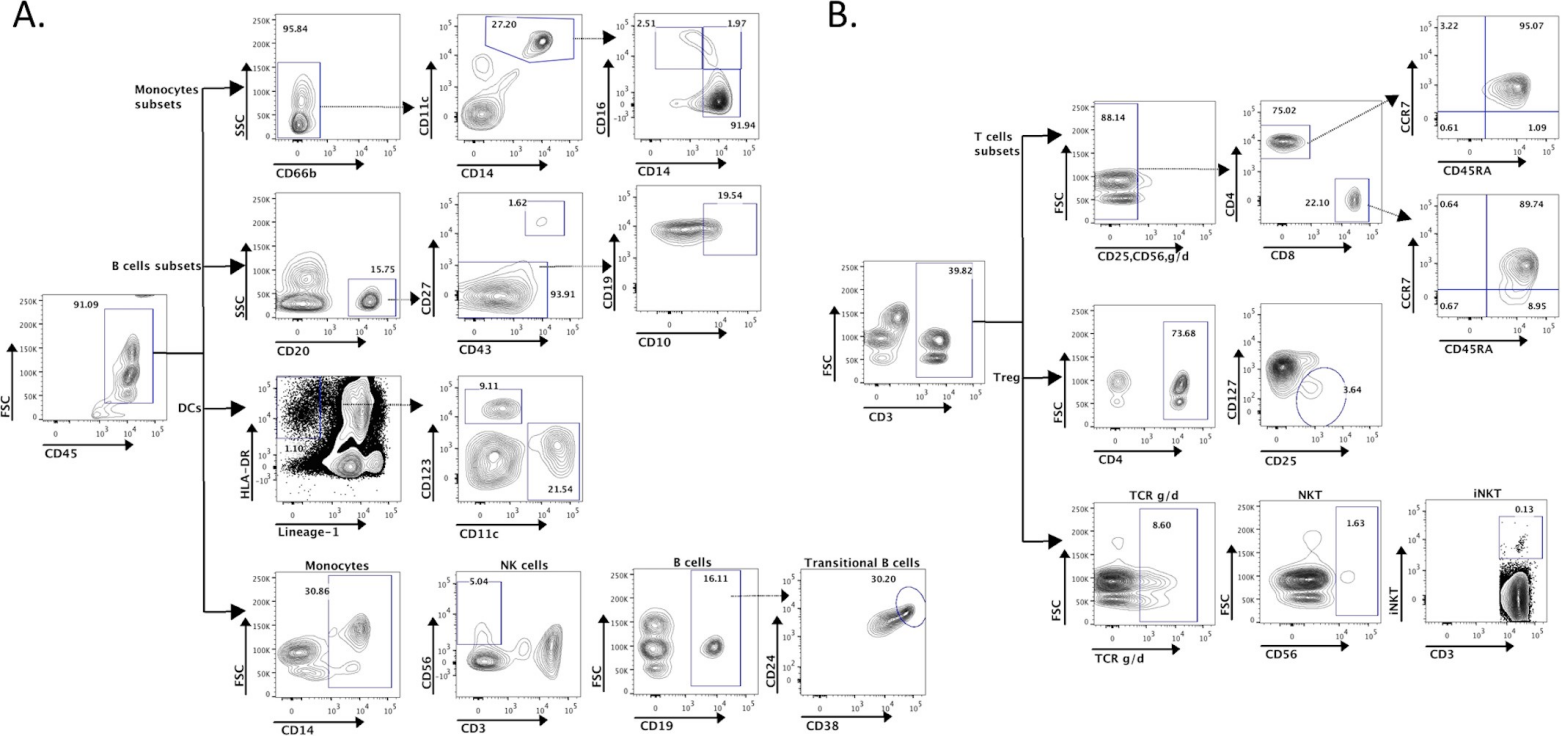
Flow cytometric-gating strategies for cell lineages from Indian and US cord blood mononuclear cells (PBMCs). After primary gating for CD45, various cell subsets were gated as shown and percentage values for each subset calculated. (A) First row: Gating for monocytes (CD45+CD66b-CD14+CD11c+), and among them, for patrolling monocytes (CD14dimCD16+), inflammatory monocytes (CD14+CD16+), and classical monocytes (CD14+CD16-). Second row: CD45+CD20+ B cells were further gated for B1 B cells (CD45+CD20+CD27+CD43+), and naïve B cells (CD45+CD20+CD27-). Naïve B cells were further gated as immature naïve B cells (CD45+CD20+CD27-CD19+CD10+). Third row: Gating for dendritic cells (DCs) (CD45+Lineage- [CD3-CD14-CD16-CD19-CD20-CD56-] HLA-DR+) and among them, for myeloid DCs (mDCs; CD45+lineage-HLADR+CD11c+CD123-) and plasmacytoid DCs (pDCs; CD45+lineage-HLA-DR+CD11c-CD123+). Fourth row: Gating for Monocytes (CD45+CD14+), NK (Natural Killer) cells (CD45+CD3-CD56+), B cells (CD45+CD19+) and Transitional B cells (CD45+CD19+CD24+CD38^bright^). (B) CD45+CD3+ cells identified as T cells. First row: Classical T cells (CD45+CD3+CD25-CD56-TCRg/d-) which were sub-divided into CD4 and CD8 cells. CD4 and CD8 T cells were further characterized using CD45RA and CCR7 as naïve (CCR7+CD45RA+) cells, central memory (CCR7+CD45RA-) and TEMRA (CD8+CCR7-CD45RA+). Second row: CD45+CD3+ T cells were further gated for putative regulatory T cells (CD45+CD3+CD4+CD25+CD127-). Third row: Gating for gamma-delta T cells (CD45+CD3+TCRg/d+), NKT cells (CD45+CD3+CD56+) and invariant NKT (iNKT) cells (CD45+ CD3+V alpha 24-J alpha 18 TCR+).

While we designed the study to have statistical power exceeding 99% for testing the primary hypotheses related to B cell subsets, we also obtained extensive information on other immune cell types (see **Fig 1** for cell subsets identified in the standard Human Immunology Project Consortium (HIPC) phenotyping panel; additional subsets were defined based on the drop-in markers added to our panel).

### Quality assurance

Harmonized protocols for collection of cord blood samples, labeling, transport, processing, and cryopreservation, were developed and shared between the two sites. All flow cytometry was performed on cryopreserved samples at only one site (THSTI). The HIPC consortium has standardized an immunophenotyping panel consisting of 5 cocktails for analysis of T cells, T regulatory cells (Treg), B cells, dendritic cell (DC)/monocyte/natural killer (NK) cells, and T helper (Th) 1, Th2, and Th17 cells [14]. Antibodies to additional cell subsets, such as gamma/delta T cells, invariant NKT cells, and B-1 cells were added to the panel, which was approved by the two collaborating sites.

### Statistical analysis

#### Primary Hypothesis

Linear-link heteroscedastic, bivariate-probit models (qlim procedure in SAS, SAS Institute, Cary, North Carolina, USA) were used to regress the joint outcome of logarithm of percentage of B-1 B cells and logarithm of IgM concentration on country, maternal age and their interaction. Results are reported for simpler model with country and maternal age as only candidate predictors where likelihood ratio test indicated interaction term did not improve fit.

#### Secondary hypothesis

Joint outcome of logarithm of percentage of immature B-2 B cells and logarithm of IgM concentration were analyzed via same methods applied to primary hypothesis.

#### Other Ig Isotopes

Each logarithmically-transformed concentration (IgA, IgG1, IgG2, IgG3, or IgG4) was regressed on country and maternal age using the icphreg procedure in SAS to allow for censored measurements below or above limits of detection.

Analysis employed interval-censored proportional hazards regression, which modeled instantaneous relative risk as a function of country and maternal age. This provided a comparisons of the distributions of the Ig isotope titers between countries, corrected for maternal age. Box plots of Fig 3 show sample distributions, including medians. Regression estimates of “survival” curves did not cross between countries for any of these Ig isotopes, thereby providing an approximate verification of the proportional hazards assumption. A satisfactory lack of fit test for assessing inclusion of interaction term (country × maternal age) was not identified. All p-values were corrected for multiple comparisons [15].

#### Other cell-subset outcomes

Separately for each cell subset, proportion of parent cells was regressed on country, maternal age, and their interaction using beta regression [16]. Goodness of fit of each regression model was verified via examination of standardized residuals [16] and plots (**S2 Fig**) of empirical quantiles versus quantiles for a beta distribution estimated from the sample of data within each country. All p-values were corrected for multiple comparisons [15].

#### Infection as outcome

Binary outcome of infection was regressed on all cell-subset and non-censored Ig-isotope variables using sparse partial least squares discriminant analysis (R package spls) with repeated (sensu [17]), five-fold cross validation and 250 resamplings of full dataset with replacement, stratified on outcome, for "bumping" [18]. Cost function for cross validation was 1 minus balanced accuracy [19] where minimum of sensitivity and specificity exceeded 0.5 and 1 otherwise. All analysis was training; sample size was too small to permit partitioning into training and testing subsamples.

Association between outcomes of breastfeeding and infection was estimated with adjustment for covariate of maternal age using a discrete, bivariate, probit analysis (qlim procedure in SAS).

Prior to each and every regression analysis, maternal age was centered and scaled as (age—A) / sample standard deviation of age, A ≈ 25–26 years. Other predictor variables were similarly centered and scaled, as appropriate. Analyses were performed in SAS and R (www.R-project.org).

## Results

### Indian and U.S. birth cohorts

We collected cord blood samples from 53 Indian and 46 U.S. infants. There were some demographic differences between cohorts, including higher maternal age (mean difference = 4.0 years, 95% confidence interval = [1.9, 6.0]) and higher birth weight (mean difference = 0.58 kg, 95% confidence interval = [0.43, 0.73]) in the U.S. participants (**Table 2)**.

**Table 2:** Baseline characteristics of participants.

	India	US	P value for difference
Father’s Age, years	n = 53	n = 41	
Mean (SD)^**1**^	28.66 (4.53)	31.39 (5.63)	
Median (IQR)	28 (26–31)	31 (29–35)	
Mother’s Age, years	n = 53	n = 45	P<0.001
Mean (SD)	24.53 (3.84)	28.49 (5.80)	
Median (IQR)	24 (22–26)	30 (23–32)	
Sex of neonate	n = 53	n = 46	P = 0.440
Female	26 (49%)	19 (41%)	
Birth weight, kg			P<0.001
Mean (SD)	2.86 (0.39)	3.43 (0.36)	
Median (IQR)	2.8 (2.6–3.1)	3.43 (3.14–3.65)	
Gestational age (days)			P<0.001
Mean (SD)	271 (8)	280 (7)	
Median (IQR)	272 (265–277)	279 (274–286)	

^**1**^SD = standard deviation; IQR = inter-quartile range; n = number of individuals

### Indo-US differences in B-1 B cells and serum IgM concentration

**Fig 2:**
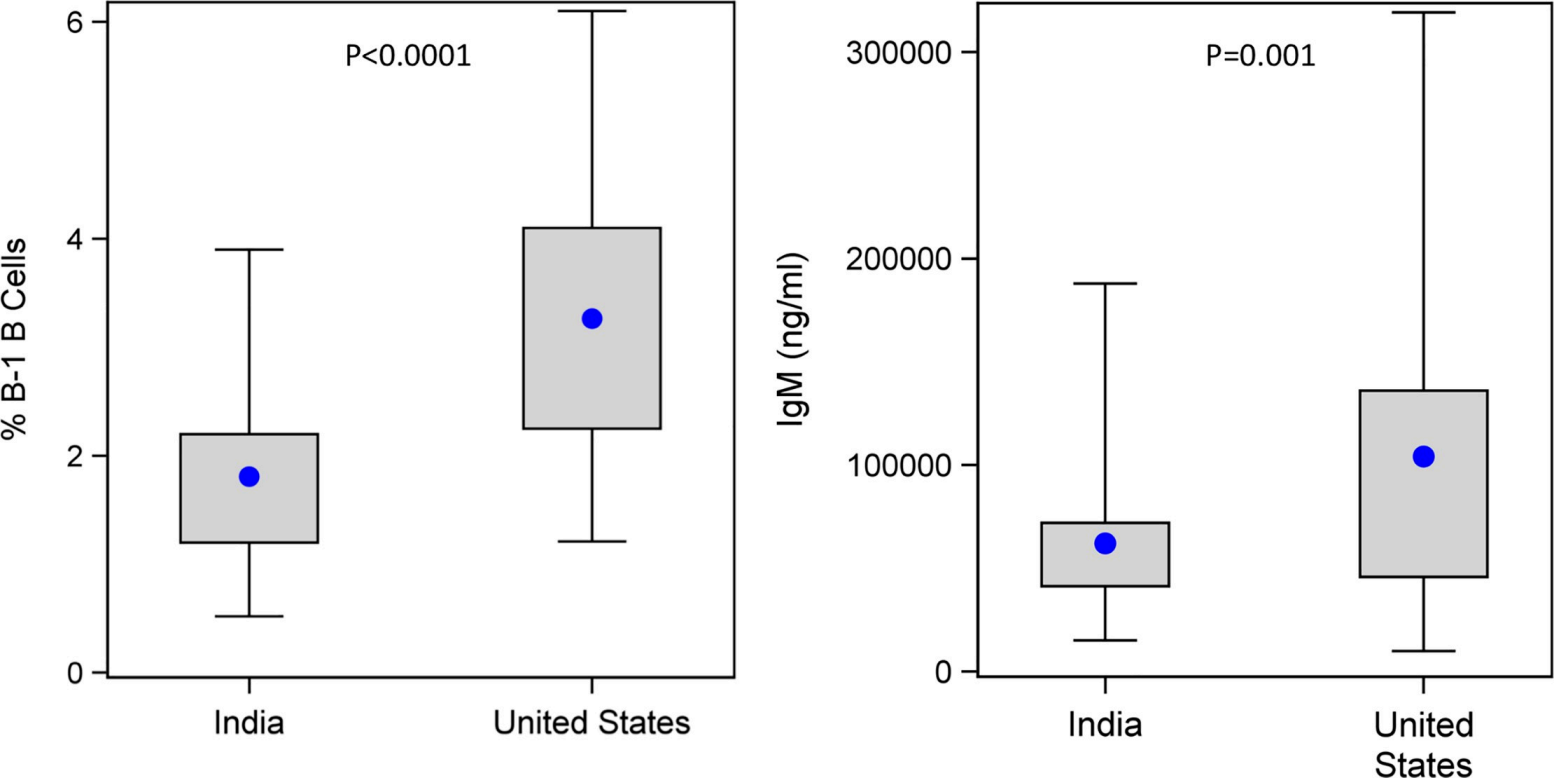
Differences in 'B-1' B cells and IgM between Indian and U.S. cohorts. B-1-like B cell percentages are shown in the left panel, with the blue dot indicating the mean, and whiskers extending from minimum to maximum values of observed data. B-1-like cells were gated as CD45+CD20+CD27+CD43+ and expressed as a percentage of total B cells (CD45+CD20+). IgM levels are shown in the right panel, as measured by Luminex assay, and expressed as ng/ml.

Our primary hypothesis was that B-1-like B cells would be lower in Indian versus U.S. cord blood, and that this would result in lower serum IgM levels as well (based on the expectation of natural antibody production by B-1 cells and the lower infection burden in the U.S.). Regression analysis was performed for both outcomes together, adjusting for maternal age because maternal age distributions differed significantly between the two cohorts (p<0.0001, **S1 Fig**). **Fig 2** provides estimates of the distributions of percentage of B-1 B cells and concentration of IgM in the two cohorts. Both were significantly higher in the United States (p<0.0001 and p = 0.001, respectively). However, there was no significant correlation between B-1 B cell percentage and IgM level (p = 0.3). Also, neither B-1 B cell percentage nor IgM level was significantly correlated with maternal age (p = 0.8 and p = 0.3, respectively). Thus, we have evidence supporting the primary hypothesis that, on average, cord blood percentage of B-1-like B cells and concentration of IgM are each elevated in the U.S. compared to India.

Using similar methods, we were unable to show a significant difference in immature B-2 B cells between the two cohorts (p = 0.6), nor any significant correlation between IgM concentration and immature B-2 B cells (p = 0.3).

### Differences in other immune features

**Fig 3:**
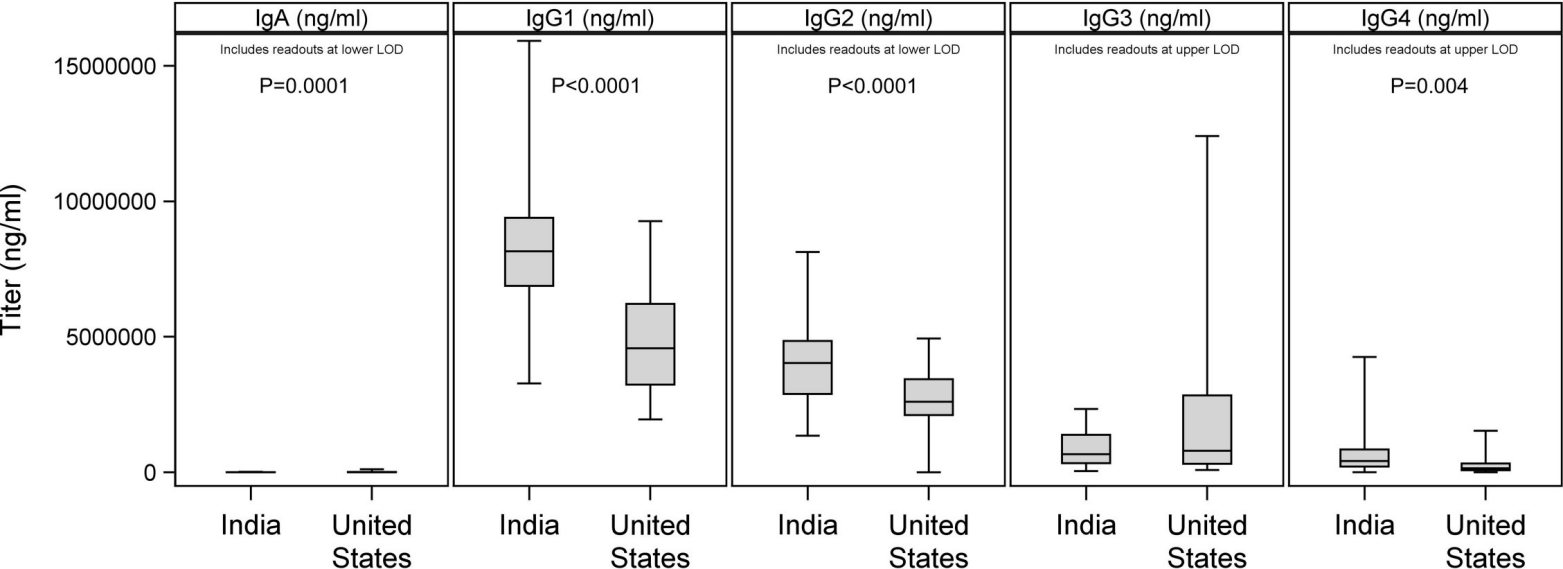
Differences in non-IgM isotypes between Indian and U.S. cohorts. Ig isotype concentrations were determined by Luminex and compared between cohorts. The horizontal bar in each plot indicates the median, with whiskers extending from minimum to maximum values of observed data. Significant differences are noted. There was one U.S. sample below the lower limit of detection (LOD) for IgG2; 10 Indian and 10 U.S. samples above the upper LOD for IgG3; 3 Indian samples above the upper LOD for IgG4; and 2 Indian and 2 U.S. samples below the lower LOD for IgA. 00.

We next tested for differences in other immune features between the Indian and U.S. cohorts. For several Ig isotypes other than IgM, we found differences: while IgA levels were significantly higher in the U.S. cohort, IgG1, IgG2, and IgG4 levels were significantly higher in the Indian cohort (**Fig 3**). IgG3 was not significantly different between cohorts.

**Fig 4:**
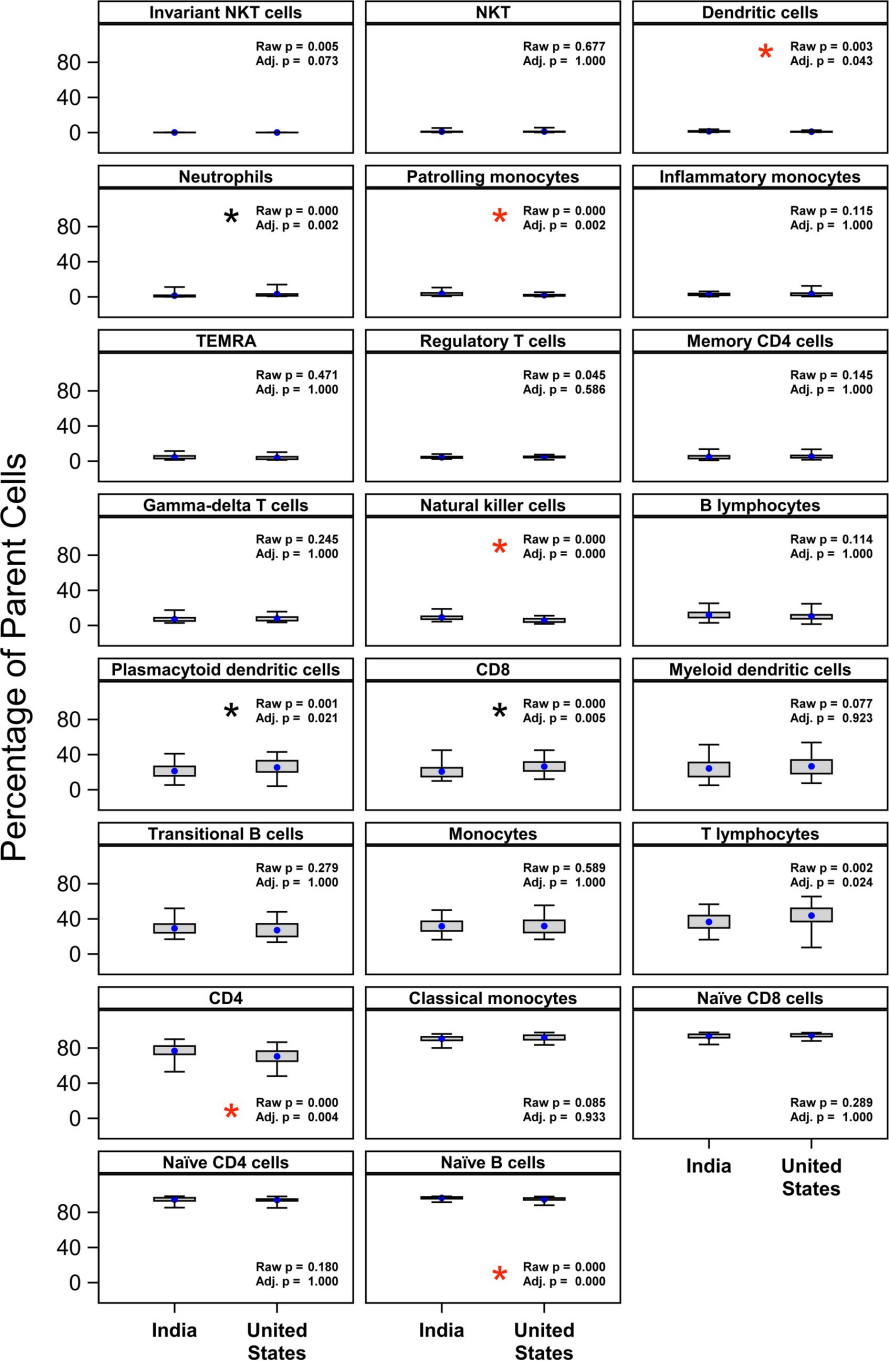
Differences in other cell subsets between Indian and U.S. cohorts. Cell subset frequencies, reported as percent of the parent subset, were regressed on country as described in the Methods. A number of significant differences in country means were detected as shown, after correction for multiple comparisons [15]. Red asterisks indicate significantly higher mean in India cohort; black asterisks indicate significantly higher mean in U.S. cohort. Neither maternal age or the interaction of country and maternal age was a statistically significant predictor of these cell-subset frequencies. Blue dots indicate the mean, with whiskers extending from minimum to maximum values of observed data.

For cell subsets other than B-1 B cells, comparisons between Indian and U.S. cohorts are shown in **Fig 4**. Subsets that were significantly higher in the Indian cohort, after correction for multiple comparisons, were dendritic cells, patrolling monocytes (CD14dimCD16+), natural killer cells, CD4+ T cells, and naïve B cells. Conversely, subsets that were significantly higher in the U.S. cohort, after multiple comparison correction, were neutrophils, plasmacytoid dendritic cells, CD8+ T cells, and total T cells. No association of outcome with age or the interaction between age and country was detected, after correction for multiple comparisons (p>0.9). Distribution of observed versus estimated sample quantiles for each cell type are shown in **S2 Fig**.

### Estimated multivariable prediction of infant infection status

**Fig 5:**
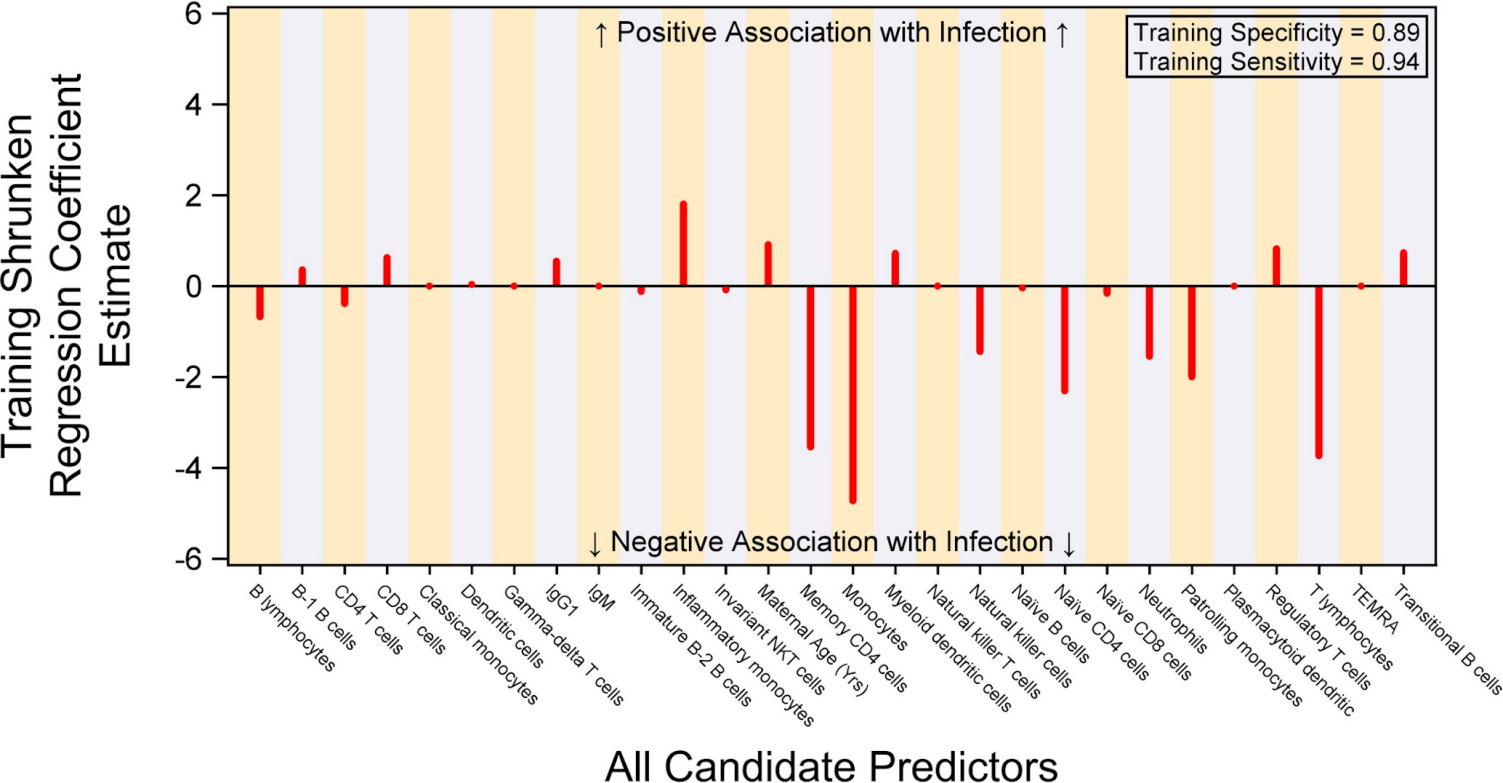
Association of cell subset frequencies with infection outcome in the Indian cohort. Regression coefficients are shown for individual features. A multivariable regression model had estimated specificity of 89% and sensitivity of 94% in predicting infant infection during the first six months of life.

Within the Indian cohort, detailed in-person follow-up was done to determine the occurrence of infections within the first six months of life **(S1 File)**. We used these data to determine whether any immune features were predictive of infection using multivariable prediction, so that each subset’s estimate is adjusted for all other subsets in the model. The results are summarized in **Fig 5**. Multiple cell subsets had strong negative predictive value for infection. Of these, monocyte frequency was the greatest negative predictor, followed by T lymphocytes, memory CD4+ T cells, naïve CD4+ T cells, patrolling monocytes, neutrophils, and natural killer cells. Subsets that were positively predictive for infection were less prominent, with inflammatory monocytes being the strongest positive predictor. Estimated sensitivity and specificity of prediction of infection status are high (≥ 0.89); but these estimates may be optimistic because they were obtained in a training sample.

## Discussion

Here we describe one of the first studies to examine immunological differences in cord blood between two geographically and ethnically different cohorts. We undertook this with a primary hypothesis related to B-1-like B cells, given their role in production of natural IgM and the importance that natural IgM may have in combatting newborn infections. Indeed, we found a significantly higher frequency of B-1-like cells and serum IgM in U.S. cord blood, as well as significant differences in other serum immunoglobulins and immune cell subsets between the two cohorts. We also found that some cell types, notably monocytes, T cells, and memory CD4+ T cells, were negative predictors of infection in the Indian cohort.

There has been some previous work reported on leukocyte subsets in umbilical cord blood across geographical areas, environments and genetic backgrounds [20–27] and there is increasing recognition of major differences across these variables [26]. However, there are few if any studies from India at the levels of granularity of subsets that we report here, and there are no direct comparisons in the literature of the kind we report here, to the best of our knowledge. Therefore, our results are difficult to interpret meaningfully in light of previous data, and are more likely to serve as a point of reference for future studies.

Our B-1-like B cell-related data will have to be interpreted cautiously, given the controversy about the validity of the CD45+CD20+CD27+CD43+ leukocytes as B-1 B cells. Initial reservations against this phenotypic identification [7] were related to the misidentification of CD3-expressing cells [8–10]. However, apart from such easily excludable mischaracterization, the subsequent major concerns addressed the possible inclusion of plasmablasts/pre-plasmablasts [28], although this is the subject of disagreement [29]. It is clear from subsequent demonstration of generation of these cells from circulating human hematopoietic stem cells [30] that the main technical issue needing resolution is the inadvertent inclusion of plasmablasts/pre-plasmablasts in the gate. Since our data address a comparison of this subset in neonatal leukocytes from neonates in India versus the USA, it is unlikely that plasmablasts/pre-plasmablasts will be significantly present, and indeed we have tested and found no plasmablasts even in our Indian neonatal populations (**S3 Fig)**. Further, we have excluded plasmablasts by using CD20+ cells rather than CD19+ cells for our B cell definition as shown above. Finally, circulating 'pre-plasmablasts', while somewhat unclear phenotypically, appear to be CD38hi, while 'B-1' B cells are CD38int [31]. We have confirmed that the subset we have identified as "B-1-like' B cells does not include any CD38hi cells (**S3 Fig)**. For these reasons, it is not unreasonable to interpret this subset in our data as B-1-like.

Higher levels of IgG1, IgG2, and IgG4 were found in the Indian cohort. Since most IgGs cross the placenta, these are probably maternally derived IgGs, possibly reflecting higher levels in maternal circulation. This in turn could reflect a higher maternal infection burden in the Indian cohort.

It is difficult to speculate on the origin or effects of cell subset differences between the two cohorts, because of the diversity of cell subset differences. Cell types that were significantly higher in Indian neonates were dendritic cells, patrolling monocytes, NK cells, CD4+ T cells, and naïve B cells; while those increased in U.S. neonates were plasmacytoid dendritic cells, CD8+ T cells and total T cells. These represent a mixture of innate and adaptive immune cells, and thus don’t suggest a clear picture of increased innate immunity or adaptive memory in one cohort versus the other.

It would be interesting to correlate these findings with data from adults. This includes correlation of immunoglobulin isotype concentrations in cord and maternal blood (and breastmilk); and changes in cord to adult blood over time. For example, the rate of accumulation of memory T cells might differ between populations.

It should be noted that our cell subset data are based on frequencies (percent of parent subset), not absolute counts. Because of this, changes in the proportion of other cell populations with the same parent could indirectly influence the results. However, we observed only one case where two populations with the same parent had significant differences between cohorts: CD4+ T cells (higher in India) and CD8+ T cells (higher in U.S.). For this case, then, it may be possible that absolute differences in one of these two populations is driving the frequency differences seen in the other.

In general, we have not established whether these differences are primarily genetic or environmental (i.e., influenced by maternal factors). That could be informed by an expanded study of U.S. neonates of Indian ancestry, in an attempt to differentiate genetics from environment. Recent publications have asserted that the frequencies of many immune cell subsets in adults are largely determined by environment [22,32].

Associations with post-natal infection suggested a prominent role for monocytes as negative predictors of infection. Total T cells as well as memory CD4+ T cells were also strong negative predictors. While it may seem counterintuitive to find T cells with a memory phenotype in cord blood, these have also been shown previously to occur at low levels [33–35]. Given the relatively small sample size and difficulties with quantifying infection risk, these studies should be repeated in a validation cohort to confirm the results. Nevertheless, the co-occurrence of multiple monocyte and T cell subsets as negative predictors of infection are intriguing they suggest the potential value of cord blood immunophenotyping to prognose risk for postnatal infection. Such testing could eventually suggest infants for whom differential post-natal follow-up might be indicated.

Because of the many possible confounding factors between India and the U.S., we limited our analysis of post-natal infection to the Indian cohort. Nevertheless, there could be confounding factors that account for some of the findings. For example, chronic herpesvirus infections could influence immune cell phenotypes, while directly contributing to the risk for other infections. In such a case, the immune cell changes could still be a biomarker of infection risk, but not a causative factor.

Additional studies linking findings like ours to potential environmental causes may provide a rationale for more intensive exposure prevention programs for toxicant/infection-exposed populations, and also suggest immunological interventions that could be helpful.

## Supporting information

S1 Fig Estimated distribution of maternal age (years) by country. Shown are kernel density estimates (Silverman 1986) by country. Despite the different distributions, significant effects of maternal age on the primary hypotheses were not seen. Ig isotypes with any values below or above detection limits were not included in this analysis. Distributions of maternal age differ between countries (p<0.0001, Kolmogorov-Smirnov two-sample test).
(DOCX)

S2 Fig Observed vs. estimated quantile distributions for cell subset frequencies in U.S. and Indian cohorts. Gray line denotes perfect agreement between observed data and fitted model.
(PDF)

S3 Fig Plasmablasts and B-1 B cells. **(A)** Lack of plasmablasts in neonatal PBMC. Representative adult and neonatal PBMC phenotyping demonstrates a population of CD38^high^CD20^-^ plasmablasts in the adult but not neonatal blood. **(B)** CD38 levels on plasmablasts vs. B-1 B cells. Plasmablasts from adult PBMC (top panel) are CD38^high^, whereas presumptive B-1 cells from adults or neonatal PBMC (middle and lower panels, gated as shown) are CD38^intermediate^.
(DOCX)

S1 File Questionnaire for follow up on infections during 6 months post-birth. (DOCX)
