# Correction: Differences in multiple immune parameters between Indian and U.S. infants

**DOI:** 10.1371/journal.pone.0214749

**Published:** 2019-03-28

**Authors:** The PLOS ONE Staff

There is an error in the first sentence of the Immunoglobulin analysis by Luminex subsection of the Materials and methods. The correct sentence is: A panel of immunoglobulins (IgG1, IgG2, IgG3, IgG4, IgM and IgA) were analysed in a Luminex 200 instrument at the laboratories of the 2 sites using the same kit from MilliporeSigma (Burlington, MA, USA) and following a standardised protocol.

There are errors in the Infection as outcome subsection of the Materials and methods; the second paragraph was not intended to be included in the published article. The complete, correct Infection as outcome subsection is as follows:

“Binary outcome of infection was regressed on all cell-subset and non-censored Ig-isotope variables using sparse partial least squares discriminant analysis (R package spls) with repeated (sensu [17]), five-fold cross validation and 250 resamplings of full dataset with replacement, stratified on outcome, for "bumping" [18]. Cost function for cross validation was 1 minus balanced accuracy [19] where minimum of sensitivity and specificity exceeded 0.5 and 1 otherwise. All analysis was training; sample size was too small to permit partitioning into training and testing subsamples.

Prior to each and every regression analysis, maternal age was centered and scaled as (age—A) / sample standard deviation of age, A ≈ 25–26 years. Other predictor variables were similarly centered and scaled, as appropriate. Analyses were performed in SAS and R (www.R-project.org). The publisher apologizes for the error.”

There are errors in the caption for Fig 3. Please see the complete, correct Fig 3 caption here.

**Fig 3:**
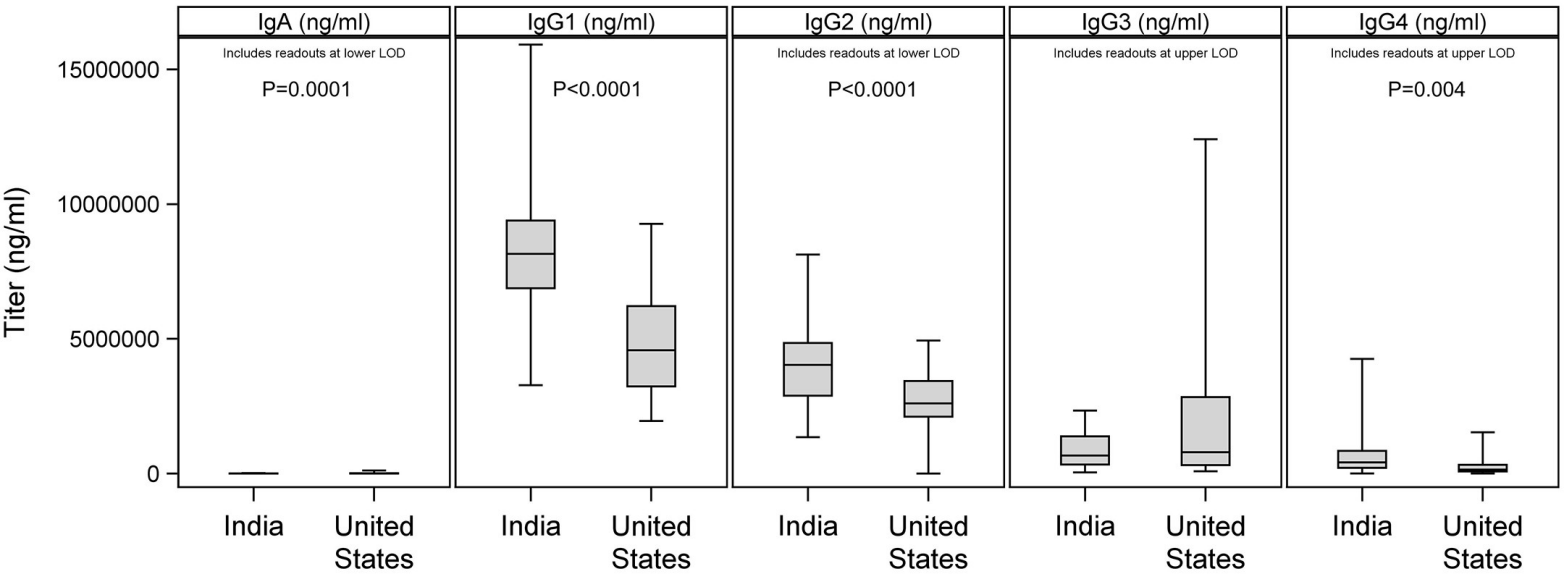
Differences in non-IgM isotypes between Indian and U.S. cohorts. Ig isotype concentrations were determined by Luminex and compared between cohorts. The horizontal bar in each plot indicates the median, with whiskers extending from minimum to maximum values of observed data. Significant differences are noted. There was one U.S. sample below the lower limit of detection (LOD) for IgG2; 10 Indian and 10 U.S. samples above the upper LOD for IgG3; 3 Indian samples above the upper LOD for IgG4; and 2 Indian and 2 U.S. samples below the lower LOD for IgA.

In Fig 4, the black asterisk is missing from the plot for T lymphocytes. Please see the correct Fig 4 here.

**Fig 4:**
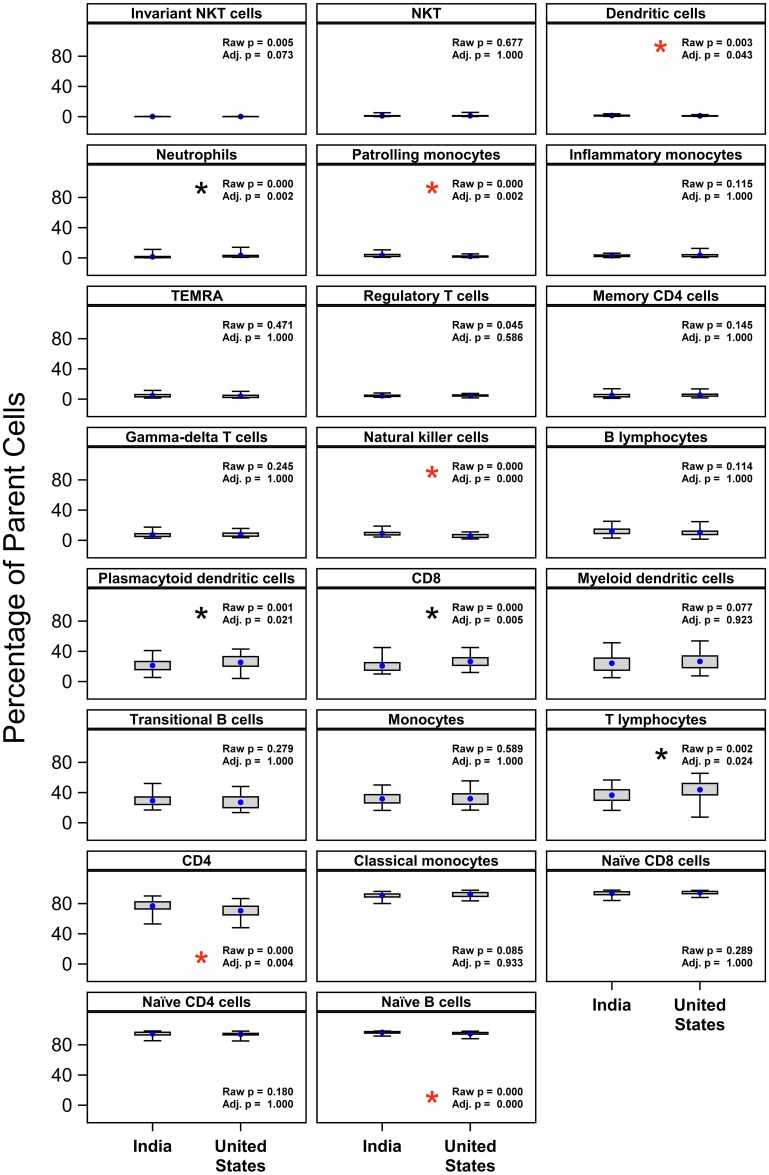
Differences in other cell subsets between Indian and U.S. cohorts. Cell subset frequencies, reported as percent of the parent subset, were regressed on country as described in the Methods. A number of significant differences in country means were detected as shown, after correction for multiple comparisons [15]. Red asterisks indicate significantly higher mean in India cohort; black asterisks indicate significantly higher mean in U.S. cohort. Neither maternal age or the interaction of country and maternal age was a statistically significant predictor of these cell-subset frequencies. Blue dots indicate the mean, with whiskers extending from minimum to maximum values of observed data.
