# Supplementary material for: Differences in multiple immune parameters between Indian and U.S. infants
Source: PLoS One. 2018 Nov 16;13(11):e0207297. doi: 10.1371/journal.pone.0207297 (PMC6239317; doi:10.1371/journal.pone.0207297)
Supplement: S1 Fig — Shown are kernel density estimates (Silverman 1986) by country. Despite the different distributions, significant effects of maternal age on the primary hypotheses were not seen. Ig isotypes with any values below or above detection limits were not included in this analysis. Distributions of maternal age differ between countries (p<0.0001, Kolmogorov-Smirnov two-sample test). (DOCX) [file pone.0207297.s001.docx]

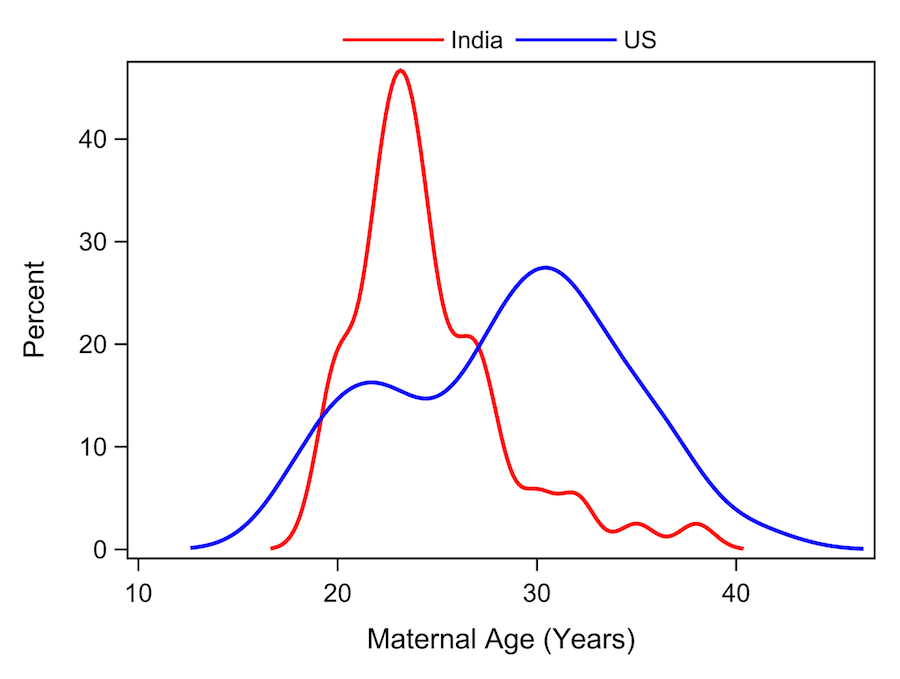


**S1 Fig.** **Estimated distribution of maternal age (years) by country.** Shown are kernel density estimates (Silverman 1986) by country. Despite the different distributions, significant effects of maternal age on the primary hypotheses were not seen. Ig isotypes with any values below or above detection limits were not included in this analysis. Distributions of maternal age differ between countries (p<0.0001, Kolmogorov-Smirnov two-sample test).
