# Supplementary material for: Differences in multiple immune parameters between Indian and U.S. infants
Source: PLoS One. 2018 Nov 16;13(11):e0207297. doi: 10.1371/journal.pone.0207297 (PMC6239317; doi:10.1371/journal.pone.0207297)
Supplement: S2 Fig — Gray line denotes perfect agreement between observed data and fitted model. (PDF) [file pone.0207297.s002.pdf]

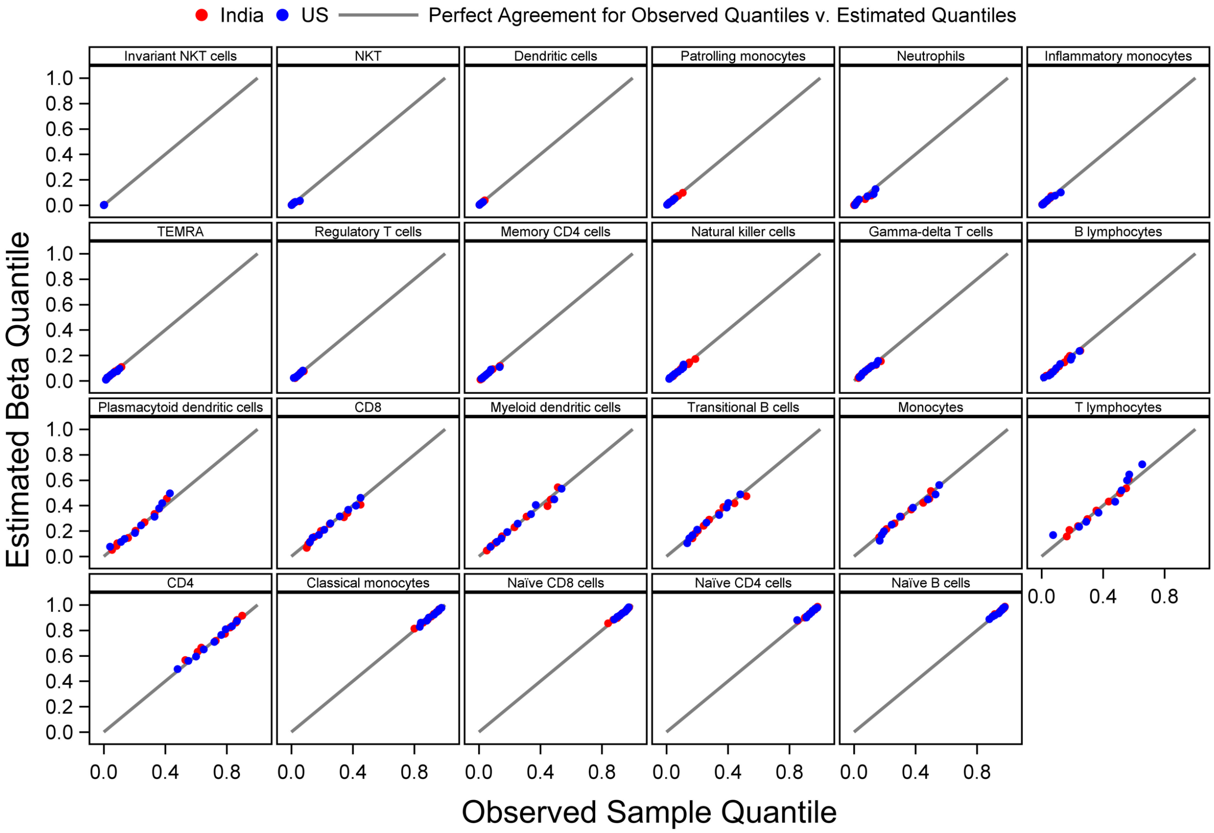

S2 Fig. Observed vs. estimated quantile distributions for cell subset frequencies in U.S. and Indian cohorts. Gray line denotes perfect agreement between observed data and fitted model.
