# Supplementary material for: Differences in multiple immune parameters between Indian and U.S. infants
Source: PLoS One. 2018 Nov 16;13(11):e0207297. doi: 10.1371/journal.pone.0207297 (PMC6239317; doi:10.1371/journal.pone.0207297)
Supplement: S3 Fig — (A) Lack of plasmablasts in neonatal PBMC. Representative adult and neonatal PBMC phenotyping demonstrates a population of CD38highCD20- plasmablasts in the adult but not neonatal blood. (B) CD38 levels on plasmablasts vs. B-1 B cells. Plasmablasts from adult PBMC (top panel) are CD38high, whereas presumptive B-1 cells from adults or neonatal PBMC (middle and lower panels, gated as shown) are CD38intermediate. (DOCX) [file pone.0207297.s003.docx]

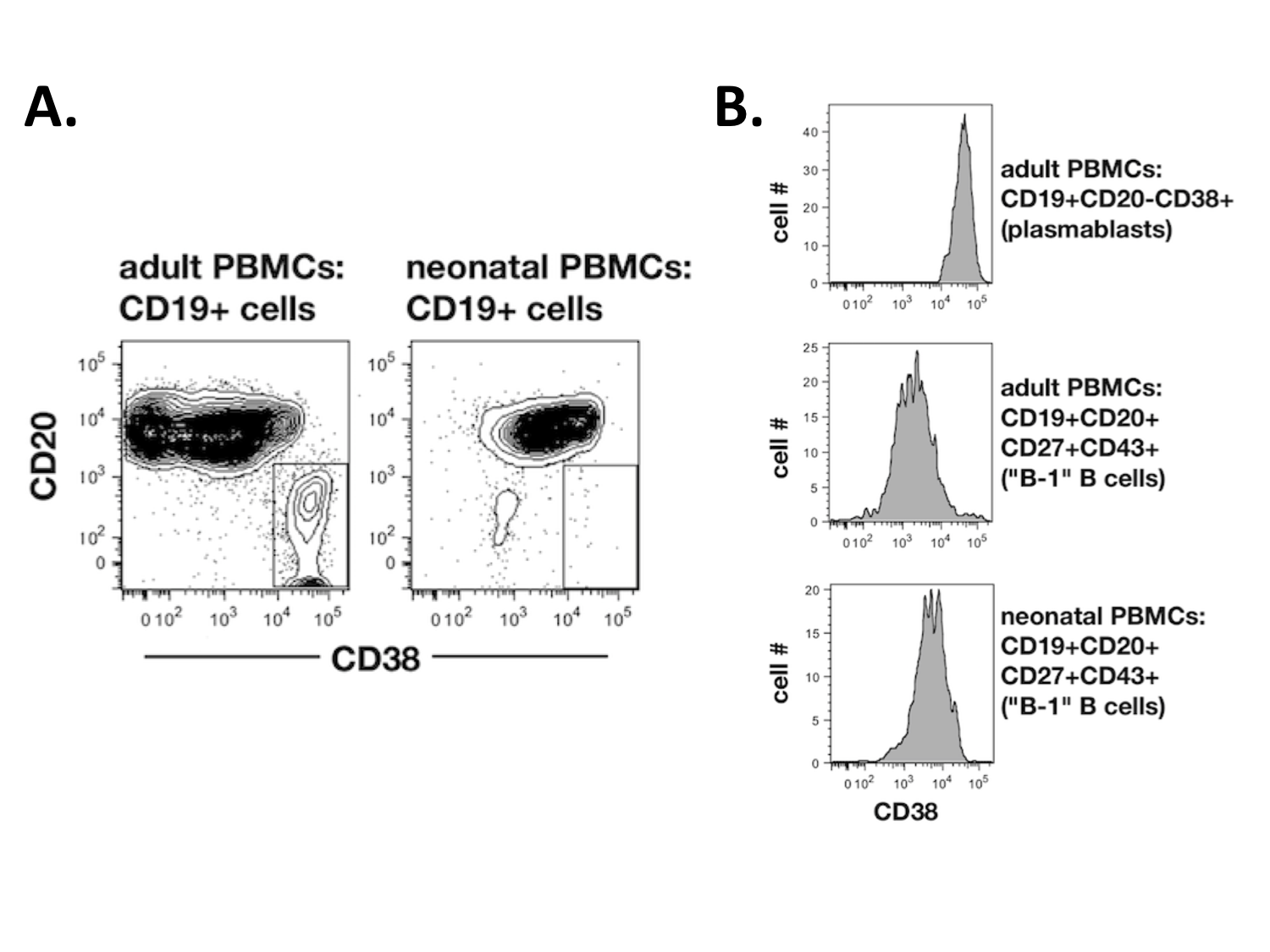


**S3 Fig. Plasmablasts and B-1 B cells. (A)** Lack of plasmablasts in neonatal PBMC. Representative adult and neonatal PBMC phenotyping demonstrates a population of CD38^high^CD20^-^ plasmablasts in the adult but not neonatal blood. **(B)** CD38 levels on plasmablasts vs. B-1 B cells. Plasmablasts from adult PBMC (top panel) are CD38^high^, whereas presumptive B-1 cells from adults or neonatal PBMC (middle and lower panels, gated as shown) are CD38^intermediate^.
