# Supplementary material for: Differences in multiple immune parameters between Indian and U.S. infants
Source: PLoS One. 2018 Nov 16;13(11):e0207297. doi: 10.1371/journal.pone.0207297 (PMC6239317; doi:10.1371/journal.pone.0207297)
Supplement: S1 File — (DOCX) [file pone.0207297.s004.docx]

**S1 File. Questionnaire for follow up on infections during 6 months post-birth.**
